# Comprehensive Cardiac Care: How Much Does It Cost?

**DOI:** 10.3390/ijerph20064980

**Published:** 2023-03-11

**Authors:** Grzegorz Kubielas, Dorota Diakowska, Michał Czapla, Bartosz Uchmanowicz, Jakub Berezowski, Izabella Uchmanowicz

**Affiliations:** 1 Department of Nursing and Obstetrics, Faculty of Health Sciences, Wroclaw Medical University, 51-618 Wroclaw, Poland; 2 Department of Health Care Services, Polish National Health Fund, Central Office in Warsaw, 02-528 Warsaw, Poland; 3 Department of Basic Sciences, Faculty of Health Sciences, Wroclaw Medical University, 50-367 Wroclaw, Poland; 4 Department of Emergency Medical Service, Faculty of Health Sciences, Wroclaw Medical University, 51-616 Wroclaw, Poland; 5 Institute of Heart Diseases, University Hospital, 50-566 Wroclaw, Poland; 6 Group of Research in Care (GRUPAC), Faculty of Health Science, University of La Rioja, 26006 Logrono, Spain; 7 University Hospital, 50-566 Wroclaw, Poland

**Keywords:** cardiac rehabilitation, comprehensive cardiac care, myocardial infarction, secondary prevention, CCMI model, myocadiac infarction

## Abstract

The benefits of coordinating care between healthcare professionals and institutions are the main drivers behind reforms to the payment and delivery system for healthcare services. The purpose of this study was to analyse the costs incurred by the National Health Fund in Poland related to the comprehensive care model for patients after myocardial infarction (CCMI, in Polish: KOS-Zawał). Methods: The analysis involved data from 1 October 2017 to 31 March 2020 for 263,619 patients who received treatment after a diagnosis of first or recurrent myocardial infarction as well as data for 26,457 patients treated during that period under the CCMI programme. Results: The average costs of treating patients covered by the full scope of comprehensive care and cardiac rehabilitation under the programme (EUR 3113.74/person) were higher than the costs of treating patients outside of that programme (EUR 2238.08/person). At the same time, a survival analysis revealed a statistically significantly lower probability of death (*p* < 0.0001) in the group of patients covered by CCMI compared to the group not covered by the programme. Conclusions: The coordinated care programme introduced for patients after myocardial infarction is more expensive than the care for patients who do not participate in the programme. Patients covered by the programme were more often hospitalised, which might have been due to the good coordination between specialists and responses to sudden changes in patients’ conditions.

## 1. Introduction

Coordinated care usually has a specific purpose, and while approaches to it may vary significantly, the overall goal is to facilitate the delivery of appropriate healthcare services in the correct order, in the proper setting and at the right time [1,2]. The benefits of coordinating care between clinicians, specialists and institutions are the driving force behind reforms to the payment and delivery system for healthcare services. It is widely believed that the better coordination of care is the remedy for the fragmented healthcare system, which can lead to improved health outcomes, a better patient experience and lower costs. However, previous studies on the impact of coordinated care programmes have yielded mixed results, so it is unclear whether the ‘promise’ of those programmes has been or will be fulfilled—especially when it comes to controlling overall medical costs [3,4].

There are few thorough assessments of the cost-effectiveness of care coordination programmes. The research in that field has generally produced contradictory results, partially due to the highly heterogeneous nature of the population and the interventions under study [3]. Some studies found that coordinating care activities can reduce visits to A&E departments and hospital readmissions, especially in the case of high-risk elderly patients; however, no formal analyses of cost-effectiveness were included in those studies [5,6,7]. On the other hand, the Community Preventive Services Task Force found sufficient evidence of the cost-effectiveness of community-based health workers’ interventions in preventing cardiovascular disease as well as preventing and treating diabetes [8]. Coordinated healthcare has become very popular in recent years because it is focused on the patient. Coordinated care providers do not divide the services, instead focusing on solving a specific health problem. Therefore, the healthcare provider focuses on solving a specific health problem, while the payer finances the whole treatment rather than the individual stages of it [9]. Acute coronary syndrome (ACS) is a broad term that covers ST-elevation myocardial infarction, non-ST-elevation myocardial infarction and unstable angina, which are very common in Poland and around the world [1]. The comprehensive care model after myocardial infarction (CCMI, in Polish: KOS-Zawał), a coordinated care programme, was introduced to improve care in patients after ACS by providing comprehensive care and cardiac rehabilitation after myocardial infarction [10,11].

The main goals of CCMI were reducing the time from hospital discharge to full revascularisation (the widening and unclogging of a narrowed blood vessel), improving access to electrotherapy (e.g., implanting cardiac pacemakers) and significantly reducing the waiting times for post-hospital cardiology consultation and cardiac rehabilitation. The programme resulted in a 29% reduction in the risk of death after myocardial infarction [10]. The purpose of this study was to analyse the costs of comprehensive care, including this cardiac rehabilitation programme, incurred by the National Health Fund in Poland.

## 2. Materials and Methods

### 2.1. Demographic Data

The analysis involved data that covered 1 October 2017 to 31 March 2020 and were collected from 263,619 patients diagnosed with first or recurrent myocardial infarction, as well as data on 26,457 patients treated during that period as part of CCMI. The demographic characteristics and the number of deaths within 365 days of the first hospitalisation in both groups of patients are shown in Table 1. Among the patients covered by CCMI, the average age was significantly lower than among those not covered by the programme (*p* < 0.001). Significantly more men were in the group covered by CCMI than in the group not covered by it (*p* < 0.0001). Mortality due to myocardial infarction was significantly higher in the patients not covered by CCMI than in those in the CCMI group (*p* < 0.0001), in terms of both the entire study group and the subgroups of women and men (*p* < 0.0001). The number of patients in the programme is lower than the baseline because not every patient treated under CCMI was hospitalised due to myocardial infarction, as reported under the programme.

### 2.2. Comprehensive Care Model after Myocardial Infarction (CCMI) Programme

The CCMI programme consisted of four modules:

◦ *Module I* included hospitalisation with conservative/invasive treatment and invasive diagnostics. It was also necessary to develop a plan of patient care and to book a coordination appointment in order to complete the module.
◦ *Module II* covered cardiac rehabilitation, which could take the form of outpatient, inpatient or hybrid care. This stage lasted a maximum of 14 days after the patient was discharged from hospital following full coronary revascularisation.
◦ *Module III* included electrotherapy introduced at any time. This enabled the implantation of a cardiac resynchronisation therapy defibrillator or an implantable cardioverter defibrillator.
◦ *Module IV* entailed specialist cardiac care lasting 12 months after the myocardial infarction.

As part of the care, patients had access to medical advice every day of the week, as well as to diagnostic tests, which could be performed 24 h a day in the cardiology department if patients’ conditions so required. Visits to the outpatient clinic were unlimited for patients covered by the programme, but a minimum of three visits were required during the 12 months of the programme. This stage ended with a care assessment that included specialist advice and laboratory tests [12].

### 2.3. Statistical Analysis

Descriptive statistics were used for data presentation. Qualitative data are shown as a number of observations with frequency distributions, while quantitative data are presented as means and standard deviations (x ± SD). A Student’s *t*-test or chi-square test was performed for comparative analysis between two independent groups. Statistical analysis was conducted using Microsoft Excel Professional 2016 (Microsoft, Redmond, WA, USA) and R software version 3.6.1 (R Foundation, Vienna, Austria).

## 3. Results

The cost-effectiveness analysis of the National Health Fund for Module I was performed on a group of 23,724 patients covered by CCMI. Table 2 shows the treatment combinations of these patients (n = 23,724) under Module I and the cost-effectiveness analysis. The most expensive method of treatment was bypass surgery (EUR 7084/person), while the lowest costs were related to conservative treatment (EUR 1364/person).

**Figure 1:**
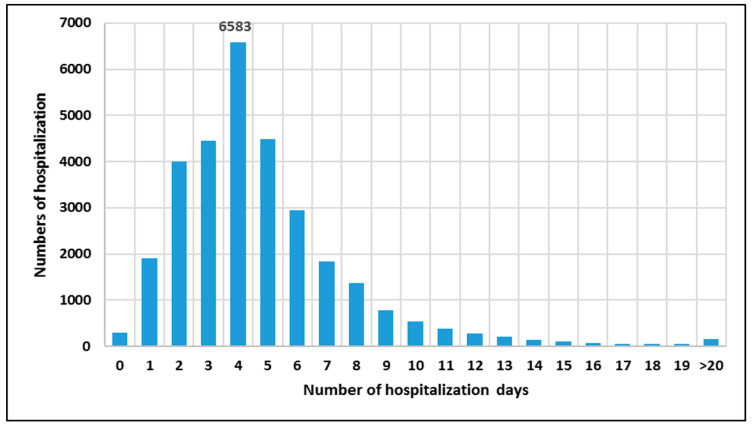
Length of hospitalisation (in days) of hospital readmissions reported for patients covered by CCMI.

Among the patients treated under CCMI, the largest number of re-hospitalisations (6583) was 4 days long, followed by 3-day and 5-day re-hospitalisations (4460 each) (Figure 1).

The total treatment costs for patients covered by CCMI (Table 3). The most expensive services were those provided under Module I, followed by those under Module II.

The total costs of treatment for patients not covered by CCMI are presented in Table 4. As with patients covered by the programme (Table 4), hospital treatment was the most expensive measure, followed by cardiac rehabilitation.

**Figure 2:**
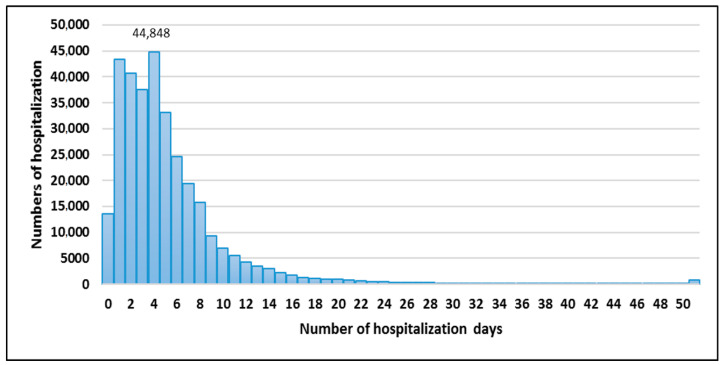
Length of hospitalisation (in days) of hospital readmissions reported for patients not covered by CCMI.

The largest number of re-hospitalisations (44,848) among patients not covered by CCMI, as with those included in the programme, was 4 days long. The second-largest group of re-hospitalisations was 1-day hospital stays (43,372), followed by 2-day hospital stays (40,745). Longer stays were the least frequent type of re-hospitalisations (Figure 2).

Table 5 presents the expenditure (in EUR) on outpatient specialist care for patients not covered by CCMI, organised by the most expensive settlement items. The largest amount of money was spent on specialist cardiac services (types 1 to 9).

In turn, Table 6 presents the expenditure (in EUR) on the rehabilitation of patients not covered by CCMI. The most expensive inpatient services were those related to cardiac rehabilitation in patients with comorbidities and the costs of cardiac rehabilitation in a day centre per person per day.

The collected data were used in a comparative analysis between the average cost of treating patients under CCMI and the average costs of treating patients outside of the programme (Table 7). There were significantly higher costs of treatment for all treatment procedures for patients covered by CCMI compared to patients not covered by the programme (*p* < 0.0001 for all).

## 4. Discussion

The coordination of care is one of several strategies [13] that integrate the care of patients with chronic or complex diseases or in need of specialist assistance [14]; the goal is to improve the coordination of care services and, thus, care planning, management or type of care. This was also the objective of the CCMI programme, the cost-effectiveness analysis of which was the topic of this study.

In 2019, in Poland, the value of healthcare services provided due to ACS and financed by the National Health Fund amounted to approximately 49% of the value of all services provided due to ischaemic heart disease (196 million euros). Hospitalisation costs amounted to 93% of the funds allocated to ischaemic heart disease, hence the conclusion that the hospital treatment of myocardial infarction consumes a significant amount of money and shows an upward trend [11].

One of the key indicators of successful care coordination is the frequency of hospital admissions (to the A&E department) or hospitalisation [15]. Furthermore, for those patients admitted to hospitals, the length of stay is a common indicator of resource utilisation. Both indicators relate to the provision of care in a more cost-effective manner. The evidence of a correlation between care coordination and a reduction in the number of hospitalisations is ambiguous. Some studies found that care coordination, as it was assumed, is associated with less A&E utilisation and fewer readmissions [16,17,18], while others found otherwise [3,19,20,21,22].

All in all, the impact that coordinating care services has on the use of hospital services and the associated costs has not been established. The results of this study indicated that the average costs of treating patients following the introduction of care coordination under CCMI (EUR 3113.74/person) were higher than the costs of treating patients outside of the programme (2238.08 EUR/person). The re-hospitalisation rate for patients under CCMI was lower than for those not covered by the programme. This may be due to programme patients being better monitored, particularly in the event of complications. It seems that the cost of coordinating care under CCMI was high, but it has not been assessed whether the introduction of coordinated care contributed to a reduced workload for general practitioners and hospital A&E departments, to which patients with deteriorating health conditions would be referred. The research indicated a 29% decline in the risk of death due to myocardial infarction (OR = 0.710; 95% CI = 0.554–0.908; *p* = 0.007) thanks to the introduction of CCMI, which is an indisputable value [9,23].

Myocardial infarction mortality remains a major challenge for modern cardiology. The implementation of the CCMI programme in 2017, a model of care based on the European Society of Cardiology guidelines, aimed to increase the quality of care provided, improve access to early cardiac rehabilitation and, most importantly, achieve long-term positive health outcomes for patients after MI. Our study demonstrated the high effectiveness of the implemented model. Patients in the CCMI group were significantly less likely to die than those in the non-CCMI group (29% reduction in risk of death), with a 39% higher total cost of care for CCMI patients compared to non-CCMI patients.

### Study Limitations

There were some limitations of the study; for example, no data were available on the workload of general practitioners or hospital A&E departments. It would be worth comparing those costs in the future, as it might reveal the financial benefits of CCMI. This study only took into account the costs incurred by the National Health Fund and of the healthcare services that were provided and billed and did not take into account the social costs resulting, for example, from the sickness absence of active patients.

## 5. Conclusions

The comprehensive care model after myocardial infarction (CCMI, in Polish: KOS-Zawał) offered to patients after myocardial infarction is more expensive than the care offered to patients not covered by the programme. Patients treated under the programme were hospitalised more often, which might have been due to the coordination between specialists and responses to sudden changes in patients’ conditions. The benefits of the programme in question outweigh the costs, including a 29% reduction in annual mortality among the affected patients, as reported by previous studies.

## Figures and Tables

**Table 1:** Demographic characteristics and number of deaths within 365 days of the first hospitalisation in the patients not covered and covered by CCMI. Descriptive data are presented as number of observations (percentage) or mean ± standard deviation (±SD).

		Patients not Covered by the CCMI Programme (n = 263,619)	Patients Covered by the CCMI Programme (n = 26,457)	*p*-Value
Gender:				
	men	160,930 (61.0)	18,090 (68.4)	<0.0001
	women	102,689 (39.0)	8367 (31.6)	
Age (mean ± SD)		68.5 ± 10.7	66.5 ± 10.8	<0.0001
Deaths:				
	men	39,955 (24.8)	2770 (15.3)	<0.0001
	women	27,358 (26.6)	1416 (16.9)	<0.0001
	total	67,313 (25.5)	4186 (15.8)	<0.0001

**Table 2:** Treatment combinations under Module I and the average cost associated with treating patients under CCMI as part of a given combination (n = 23,724). The number 0 means that the treatment procedure was not performed, while the number 1 means that the procedure was performed within the combination of treatment.

Invasive Therapy in Acute Coronary Syndrome	Conservative Treatment	Invasive Diagnostics in Acute Coronary Syndrome	Bypass	Angioplasty	Number of Patients (N)	Frequency (%)	Average Cost of Treatment (EUR/Person)
1	0	0	0	0	18,694	78.8	3410
1	0	0	0	1	1993	8.4	4820
0	0	1	0	0	1566	6.6	1540
0	0	0	0	0	380	1.6	530
0	0	0	1	0	308	1.3	7080
0	0	1	1	0	237	1.0	7940
1	0	0	1	0	166	0.7	9280
0	1	0	0	0	102	0.4	1360
0	0	0	0	1	95	0.4	2020
1	0	1	0	0	84	0.4	4860
1	1	0	0	0	43	0.2	3870
0	0	1	0	1	23	0.0	3370
0	1	1	0	0	8	0.0	2020
1	0	1	0	1	7	0.0	5870
1	0	1	1	0	6	0.0	11,090
0	0	0	1	1	6	0.0	7520
0	1	0	1	0	3	0.0	9040
1	1	0	0	1	2	0.0	7900
1	0	0	1	1	1	0.0	10,270

**Table 3:** Total costs of treatment (in EUR) of patients covered by CCMI.

Range of Treatment by Module	2017 (October–December)	2018	2019	2020 (January–March)	Total
Module I (invasive diagnosis, conservative treatment or invasive treatment)	4,008,915	24,391,894	36,312,400	9,157,130	73,870,339
Module II (cardiac rehabilitation)	313,373.50	4,435,751	7,181,619	2,068,638	13,999,381
Module III (electrotherapy)	21,472.66	826,169.10	1,388,950	333,334.10	2,569,925
Module IV (specialist cardiac care)	3204.08	283,141.30	807,419.80	257,856.10	1,351,621
Other	66,549.23	427,432.10	1,922,287	741,783	3,158,052
Total	4,413,514.22	30,364,387.55	47,612,675.85	12,558,741.39	94,949,319.02

**Table 4:** Total costs of treatment (in EUR) of patients not covered by CCMI.

Range of Treatment	2017	2018	2019	2020	Total
Hospital treatment	62,415,229.21	232,282,368.50	232,642,758.50	54,766,785.89	582,107,142.10
Outpatient specialist care	681,057.15	2,671,317.57	2,807,314.22	648,139.19	6,807,828.13
Rehabilitation	8,035,026.25	31,878,890.19	33,201,802.37	7,461,458.86	80,577,177.67
Total	71,131,312.61	266,832,576.20	268,651,875.10	62,876,383.94	669,492,147.90

**Table 5:** Expenditure (in EUR) on outpatient specialist care for patients not covered by CCMI, organised by the most expensive settlement items.

Settlement Range	Name of the Settlement Item	Number of Consultations (n)	Total Value of Services Settled (in EUR)
Nuclear medicine exams	Radioisotope myocardial perfusion scan, SPECT or gated SPECT technique	6251	619,715.72
Cardiology services	Specialist services	270,966	3,994,774.19
Cardiology services—first-time services	First-time services	39,206	1,111,296.80
Cardiac surgery services	Specialist services	4232	51,476.06
Computed tomography	Angiography	6737	880,240.95
Magnetic resonance imaging	Cardiac morphology—functional and morphological, with and without contrast enhancement	228	51,079.80
Total		327,620	6,708,583.52

**Table 6:** Expenditure (in EUR) on the rehabilitation of patients not covered by CCMI.

Settlement Range	Number of Rehabilitation Services	Total Value of Services Settled (in EUR)
Cardiac rehabilitation in an outpatient centre, per person per day	584,568	8,801,312.63
Cardiac hybrid telerehabilitation at home, per person per day	8912	145,419.47
Inpatient cardiac rehabilitation with comorbidities	103,154	68,282,369.41
Cardiac rehabilitation in hospital—category I	3242	1,310,609.64
Cardiac rehabilitation in hospital—category II	1035	257,511.98
Cardiac rehabilitation in a rehabilitation facility, for patients with comorbidities	2917	1,711,926.05
Cardiac rehabilitation in a rehabilitation facility—category I	201	64,078.29
Cardiac rehabilitation in a rehabilitation facility—category II	31	3950.20
Total	704,060	80,577,177.67

**Table 7:** Comparison of the average costs of treating patients under CCMI compared to those not included in the programme, from 2017 to 2020. Descriptive data are presented as mean ± standard deviation (±SD).

Range of Treatment	Average Cost of Treatment (EUR/Person)		*p*-Value
	Patients not Covered by CCMI (n = 263,619)	Patients Covered by CCMI (n = 26,457)	
Hospital treatment/module I	2208.14 ± 1273.30	2792.09 ± 1384.71	<0.0001
Outpatient specialist care/module IV	25.82 ± 17.39	51.09 ± 36.14	<0.0001
Rehabilitation/modules II + II	305.66 ± 154.16	626.27 ± 432.65	<0.0001
Total	2539.62 ± 1444.85	3469.45 ± 1853.50	<0.0001

## Data Availability

All relevant data are included within the article. If necessary, it is possible to contact the corresponding author to request additional materials.
